# The use of MRI apparent diffusion coefficient (ADC) in monitoring the development of brain infarction

**DOI:** 10.1186/1471-2342-11-2

**Published:** 2011-01-06

**Authors:** Jian-Min Shen, Xian-Wu Xia, Wu-Gen Kang, Jian-Jun Yuan, Liang Sheng

**Affiliations:** 1 Department of Medical imaging, Taizhou municipal hospital, Taizhou Medical College, Taizhou 318000, PR China

## Abstract

**Background:**

To study the rules that apparent diffusion coefficient (ADC) changes with time and space in cerebral infarction, and to provide the evidence in defining the infarction stages.

**Methods:**

117 work-ups in 98 patients with cerebral infarction (12 hyperacute, 43 acute, 29 subacute, 10 steady, and 23 chronic infarctions) were imaged with both conventional MRI and diffusion weighted imaging. The average ADC values, the relative ADC (rADC) values, and the ADC values or rADC values from the center to the periphery of the lesion were calculated.

**Results:**

The average ADC values and the rADC values of hyperacute and acute infarction lesion depressed obviously. rADC values in hyperacute and acute stage was minimized, and increased progressively as time passed and appeared as "pseudonormal" values in approximately 8 to 14 days. Thereafter, rADC values became greater than normal in chronic stage. There was positive correlation between rADC values and time (P < 0.01). The ADC values and the rADC values in hyperacute and acute lesions had gradient signs that these lesions increased from the center to the periphery. The ADC values and the rADC values in subacute lesions had adverse gradient signs that these lesions decreased from the center to the periphery.

**Conclusion:**

The ADC values of infarction lesions have evolution rules with time and space. The evolution rules with time and those in space can be helpful to decide the clinical stage, and to provide the evidence in guiding the treatment or judging the prognosis in infarction.

## Background

Brain infarction leads to high mortality and disability [1]. The post-infarction treatments available were largely dependent on the timing after infarction to determine the stages of brain damage [2]. The fast and accurate radiological diagnosis could provide important reference in judging the timing and development stages of brain infarction. In recent years, the diffusion weighted imaging (DWI) based on echo planar imaging (EPI) has emerged to be applicable in early diagnosis of brain infarction [3-9]. The present study investigated the changes of apparent diffusion coefficient (ADC) and relative ADC (rADC) values in different timing points after brain infarction as well as different brain regions to understand if ADC value could be instructive in clinical diagnosis of brain infarction progression and treatment.

## Methods

### Clinical data

98 cases (55 male, 43 female, ages 41-76 years with an average of 65) of brain infarction patients were diagnosed and verified with radiology data. 117 MRI and DWI examinations were performed: 12 hyperacute (< 6 hours post brain infarction), 43 acute (7-24 hours), 29 subacute (2-7 days), 10 steady (8-14 days), and 23 chronic (> 15 days) infarctions. The ethics approval was obtained by Taizhou Medical college committee of clinical research, and written form approvals were obtained from patients.

### MRI and DWI scanning

Siemens NOVUS 1.5 superconducting MR scanning system was used. Routine MRI was firstly used to define T1W1 and T2W1: T1W1 was using spin echo sequences (SE) with TR/TE of 350 ms/8 ms; T2W1 was using turbo spin echo sequences (TSE) with TR/TE = 2500 ms/9 ms; the acquisition matrix is 218 × 256. For DWI scanning, single time activated SE-EPI sequences was used with following parameters: TR3300 ms, TE94 ms, acquisition matrix 128 × 128, FOV 230 mm × 230 mm, with diffusion gradient in x, y and z dimensions, and acquire images at b = 0 and 1000; the ADC pictures were automatically built. All scanning sections were 7 mm thick with 2 mm distance to each other.

### Image analysis

Four regions of interest (ROI) were selected from central, near central, near edge, edge of infracted areas on ADC figures according to T1W1 and T2W1, with 5 pixels for each ROI to compute the average ADC value of the whole infracted region. The rADC value is given by: rADC = (average ADC value in infracted side/average ADC value in heath side) × 100%. Sulcus and ventricle were avoided in ADV value measurement.

### Statistics

The statistics were performed with SPSS12.0 software. A significant difference was considered when P < 0.01 in T test.

## Results

### The average ADC and rADC in brain infracted regions change with time

**Figure 1:**
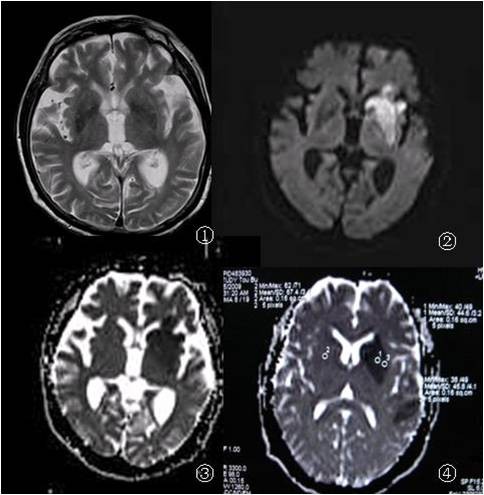
**1-3 were taken from one super acute case patient, 4 hours after brain infarction**. 1 showed the T2W1 image, which failed to diagnose the infracted region; 2 showed DWI images, with clear infracted region in basal ganglia; 3 is ADC image, showing lower ADC value in infracted region. 4 is acute case 7 hours after infarction, with lower ADC value in the center of infracted region.

**Figure 2:**
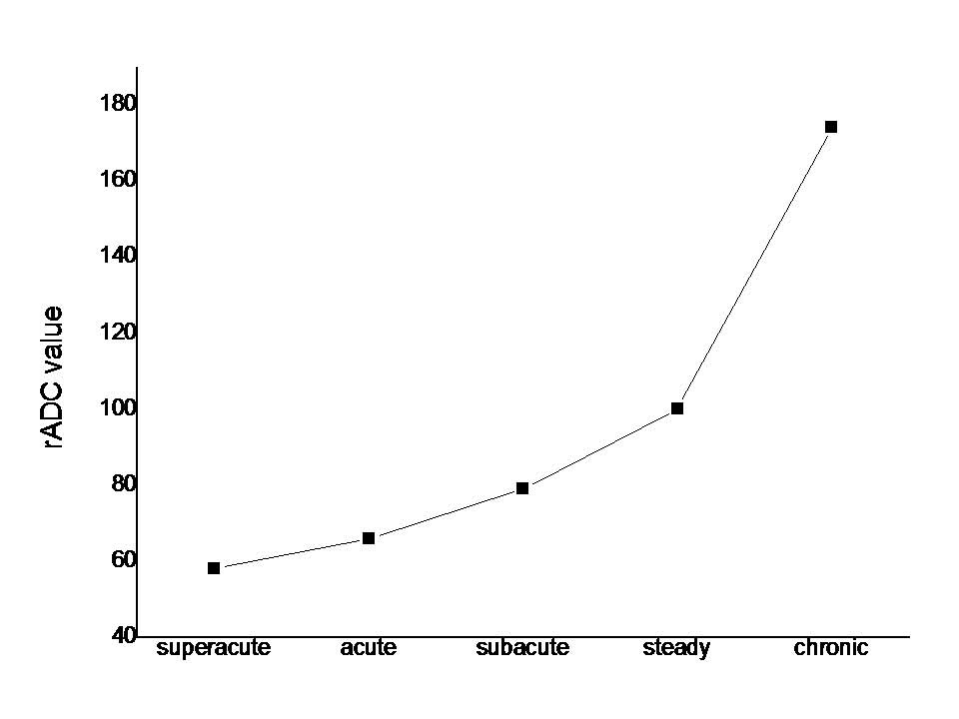
**rADC values (%) change in different time period after brain infarction**.

The average ADC values in brain infracted area and contralateral (unaffected) areas as well as the average rADC values from 117 scanning were shown in table 1. The results suggested in the super acute and acute cases the average ADC values significantly decreased and is lower than the contralateral side (Figure 1); The average rADC values were 58% and 66% for super acute and acute cases, respectively, without significant difference (P > 0.01). In subacute cases, the average ADC and rADC values were higher in compared to hyper acute and acute cases (P < 0.01), with the average rADC value of 79%. The ADC value in the infracted area came back to normal range, showing the "false normal" phenomenon. In the chronic cases the average ADC values in infracted regions were higher than the contralateral side, with an average rADC value of 174%; both values were significant different from other three time periods (P < 0.01) (Table 1, Figure 2).

**Table 1:** ADC and rADC value in different time points after brain infraction

Time phase	Number of cases	ADC valuen	rADC value (*x*±*s*)(%)
superacute(< 6 h)	12	30~63	58 ± 6.75
acute(7~24 h)	43	49~78	66 ± 5.36
subacute(2~7 d)	29	60~90	79 ± 4.83
steady (8~14 d)	10	87~108	100 ± 5.43
chronic (> 15 d)	28	110~219	174 ± 33.47

The ADC and rADC values vary within brain infracted region

In the infracted region of 12 super acute and 43 acute cases, the ADC and rADC values were unevenly distributed; they increase from the center to the edge, forming a "gradient" (Figure 1); for example, the average rADC value in the center is 61%, which was 78% in the edge. In contrast to this, the 29 subacute cases showed reversed gradient - that is, the ADC and rADC values decreased from center to the edge regions, which were 85% and 66%, respectively.

## Discussion

DWI is currently the only imaging method to investigate diffusion activities of water molecules in vivo; ADC reflects the diffusion speed of water molecules, and the fast diffusion of water molecules could be revealed with a larger ADC value with lower signals of DWI images. Therefore the changes of ADC value could reflect the water molecule diffusion status in post brain infarction periods [8,10-12]. In superacute and acute brain infarction periods, the slowdown of water molecule diffusion could be explained by the cellular edema: following ischemia, the outflow of intracellular potassium ions and the inflow of calcium, sodium as well as water caused the swelling of the cells. The water molecules inside the cells were however bounded by cell membrane and organelles, and therefore diffuse much slower, causing the lower ADC values. Because of the relative balance in total amount of water in the acute phase after brain infarction, T2W1 often show no change even that the ADC value decreased, as found in 12 super acute cases in present manuscript - which suggested that the accuracy of using ADV value in diagnosis of brain infracted regions is 100% in our hand. This cannot be reach in routine MRI images. To normalize the ADC value and eliminate the individual differences, the present study measured the ADC value in the contralateral side to compute the rADC value [13,14], which could be useful in comparative studies, even among individuals across countries.

In present study we showed that the average ADC and rADC value change with time period post-brain infarction, which is caused by pathophysiological changes following brain damages. In superacute and acute cases the rADC reached the lowest point, and began to increase in subacute cases, and appeared to be "normal" between 10-14 days after brain infarction happened. This could be explained by two possibilities: first, vessel-sourced edema caused injury to endothelial cells 5-6 hours after brain infarction, and exacerbated in subacute phase, leading to enhanced permeability of blood brain barrier, and increased movement of many molecules including water molecules in extracellular spaces; second, the cell membrane broken and released intracellular water molecules [15]. In chronic phase the infracted brain tissues liquidize and were replaced by cerebrospinal fluid, which has a free movement of water molecules and the high ADC value when compared with brain tissues, leading to a rADC value more than 100%. Given the fact the ADC and rADC values vary during the post brain infarction period, we suggest that the combined analysis of ADC, rADC and routine MRI images could be important and useful in clinical diagnosis of the progression after brain infarction.

The other interesting point is the spatial distribution of ADC and rADC values within infracted region. In superacute and acute cases ADC values increase from the center to the side areas, suggesting that the decreases of ADC values could reflect the severity of the tissue damage. The surrounding regions of brain infarction area were considered to be plastic and could be re-perfused in proper conditions, or infracted if untreated [16]. The gradient of ADC value thus provided a chance to diagnose the surrounding sensitive regions, to follow the efficacy of treatment and prediction of the recovery. In subacute phase the reversed gradient suggested the liquidation of infracted tissues from the center, and brought the increased ADC value. The "false normal" phenomena would appear in the late phase of subacute patients, and during this period the reperfusion could not save the brain tissue from necrosis. Moreover, this would point the possibility to use ADC values to predict the recovery into normal tissue or final degeneration after the intervention in patients with brain infraction [17].

## Conclusion

In summary, the present study showed that in brain infracted regions the ADC and rADC values change both temporally and spatially, reflecting the progression of infarction and tissue damage severity. The adoption of these values in clinical diagnosis and treatment of brain infarction patients could be helpful and instructive.

## Competing interests

The authors declare that they have no competing interests.

## Authors' contributions

all authors participated in the design of the experiment; JMS provided the funding and analyzed the data; all authors participated in the writing of the manuscript.

## Pre-publication history

The pre-publication history for this paper can be accessed here:

http://www.biomedcentral.com/1471-2342/11/2/prepub
